# A Slower-Progressing TDP-43 rNLS8 Mouse Model for ALS: Implications for Preclinical and Mechanistic Studies

**DOI:** 10.1007/s12017-025-08871-z

**Published:** 2025-08-18

**Authors:** Cyril Jones Jagaraj, Prachi Mehta, Julie Hunter, Julie D. Atkin

**Affiliations:** Macquarie Medical School, Faculty of Medicine, Health and Human Sciences, MND Research Centre, Macquarie University, 75 Talavera Road, Sydney, NSW 2109 Australia

**Keywords:** ALS, TDP-43 rNLS8 mice model, Longer disease course

## Abstract

Amyotrophic lateral sclerosis (ALS) is a fatal neurodegenerative disease characterised by motor neuron degeneration, muscle weakness, paralysis, and eventual death, with TAR DNA-binding protein 43 (TDP-43) pathology observed in almost all cases. Mouse models based on TDP-43 are thus essential for studying ALS and developing therapeutic approaches. The TDP-43 rNLS8 mouse model expresses a human TDP-43 transgene with a mutated nuclear localization sequence (hTDP-43 ΔNLS), but this is normally suppressed by the presence of doxycycline (Dox). Disease is initiated by removal of Dox, which replicates key ALS features, including TDP-43 pathology, neuromuscular junction denervation, motor neuron loss, and reduced survival. However, this model has a rapid disease progression which limits its use for extended preclinical studies and investigation of early disease mechanisms. To overcome these limitations, we explored whether maintaining low Dox concentrations in the diet (10–20 mg/kg) could slow disease progression. Our findings demonstrate that this approach significantly reduced hTDP-43 ΔNLS expression (up to 4.8-fold), which delayed disease onset by four weeks. Disease progression, assessed by rotarod performance, grip strength, and neurological scores, was extended from six to 15 weeks, with a threefold increase in survival. Despite slower progression, at the end stage, mice displayed similar levels of neuroinflammation, motor neuron loss, as Dox off mice. These findings highlight slower-progressing TDP-43 rNLS8 mice as a robust model for preclinical and early disease mechanism studies.

**Supplementary Information:**

The online version contains supplementary material available at 10.1007/s12017-025-08871-z.

## Introduction

Amyotrophic lateral sclerosis (ALS) is a fatal neurodegenerative disease characterised by the presence of pathological forms of TAR DNA-binding protein 43 (TDP-43) in the brain and spinal cord in almost all cases (97%) (Neumann et al., 2006). TDP-43 pathology involves its aberrant mislocalisation to the cytoplasm, with aggregation, fragmentation, ubiquitination, and hyper-phosphorylation of serine residues at 403/404 and 409/410 (Arai et al., 2006; Neumann et al., 2006; Suk & Rousseaux, 2020). Additionally, approximately 50% of frontotemporal lobar degeneration (FTLD) cases also exhibit TDP-43 pathology (known as FTLD-TDP) (Cairns et al., 2007; Neumann et al., 2021; Weihl et al., 2008), and ALS and FTLD-TDP are considered to form a continuous disease spectrum (Neumann et al., 2021; Shen et al., 2023; Weihl et al., 2008). Most ALS cases arise sporadically, and only 10% of ALS and 30% of FTLD-TDP cases are familial (Hasegawa et al., 2008; Jagaraj et al., 2020, 2024; Shen et al., 2023). TDP-43 pathology is also present in approximately 19–57% of Alzheimer's disease (AD) cases and 75% of severe AD cases (Josephs et al., 2014; Uryu et al., 2008), and the prevalence of TDP-43 in AD increases with age (Jo et al., 2020). These findings highlight the importance of TDP-43 pathology in neurodegeneration.

Most mouse models developed for ALS rely on overexpression of proteins bearing genetic mutations present in only a small minority of patients (Liscic, 2017). This includes transgenic mice expressing mutant superoxide dismutase 1 G93A (SOD1^G93A^), which have been widely used for over two decades (Tu et al., 1997). Whilst they display phenotypes reminiscent of ALS, these mice do not display the TDP-43 pathology present in most cases (Mackenzie et al., 2007). Hence, therapies developed in mice models with TDP-43 pathology may be preferable and more effective for developing treatments applicable to most ALS cases.

Over twenty TDP-43 mouse models are now available, including TDP-43 rNLS8 (Walker et al., 2015), which involves transgenic overexpression of human TDP-43 (h-TDP-43) with a mutated nuclear localization sequence (h-TDP-43 ΔNLS), driven by the human neurofilament heavy chain (NEFH) promoter (Walker et al., 2015). In these animals, extensive accumulation, aggregation, and hyperphosphorylation of h-TDP-43 in the neuronal cytoplasm result in both the brain and spinal cord, reminiscent of the TDP-43 pathology present in human ALS (Walker et al., 2015). This mouse has advantages compared to other TDP-43 models because it displays TDP-43 pathology with  neuronal loss, muscle atrophy, motor phenotype, and premature death. Expression of h-TDP-43 ΔNLS is inducible, controlled by tetracycline transactivator protein (tTA), which is regulated by doxycycline (Dox, 200 mg/kg). When Dox is removed from the diet (‘Dox off’), h-TDP-43 ΔNLS expression is activated, whereas when Dox is added to the diet (‘Dox on’), expression is suppressed (Walker et al., 2015). Another key aspect of this model is its ability to replicate the timeline of development of TDP-43 pathology, neuromuscular junction denervation, and spinal cord and cortical motor neuron loss, resembling the progression of ALS in humans (Walker et al., 2015). TDP-43 pathology and a motor phenotype manifest two weeks after the removal of Dox, marking the disease onset stage. By four weeks off Dox, the mice exhibit muscle denervation and cortical atrophy (early disease stage). By six weeks off Dox, they begin to display a severe motor phenotype and approximately 30% loss of motor neurons in the spinal cord (late disease stage), and they reach end-stage (humane endpoint) at ten weeks off Dox (Walker et al., 2015). However, re-introduction of Dox (200 mg/kg) to the diet results in re-suppression of h-TDP-43 ΔNLS expression and subsequent clearance of TDP-43 pathology (Walker et al., 2015). This leads to neuronal preservation, rescue of motor impairment, and an extension of lifespan, even after significant neurodegeneration and motor dysfunction have already taken place (Walker et al., 2015).

Whilst several therapeutic interventions for ALS have been previously examined in TDP-43 rNLS8 mice (Luan et al., 2023; Riemenschneider et al., 2023; Spiller et al., 2019; Tsitkanou et al., 2022; Wright et al., 2021), they have had limited success. A significant contributing factor is the high levels of h-TDP-43 ΔNLS overexpression, which results in rapid disease progression: mice reach late disease stages only 5–6 weeks after Dox removal. Recent studies have also shown that high levels of overexpression of TDP-43 generally in mice produce toxic gain-of-function effects that may not be relevant to human ALS (Carmen-Orozco et al., 2023; Carmen-Orozco et al., 2024). The accelerated disease phenotype in TDP-43 rNLS8 mice therefore creates a narrow therapeutic window that can be difficult to effectively evaluate potential treatments (de Boer et al., 2021). Hence, the rapid disease course may compromise preclinical studies using this model; the short timeframe between disease onset and severe symptoms may limit the opportunity for therapeutic interventions to demonstrate meaningful effects, as well as compromise authentic exploration of disease mechanisms (Jo et al., 2020). Consequently, translating findings from this model into clinically relevant therapeutic strategies may be problematic, emphasising the need for alternative approaches. Transgenic rNLS8 mice with lower levels of TDP-43 expression, with a much slower disease onset and progression, may be better suited for long-term therapeutic studies and to explore early disease mechanisms.

In this study, we examined whether we could delay disease onset and progression in TDP-43 rNLS8 mice using low concentrations of Dox (10 mg/kg and 20 mg/kg) in the diet, rather than removing it completely. We demonstrate that maintaining Dox at these much lower concentrations (20- and 10-fold respectively) resulted in a significantly slower disease course than previously documented (Luan et al., 2023; San Gil et al., 2024; Spiller et al., 2016; Wright et al., 2021). This approach led to up to almost fivefold less expression of h-TDP-43 ΔNLS in the cytoplasm of neurons, leading to up to a fourfold delay in both disease onset and progression, and almost threefold extension of survival. Hence, using this strategy, the TDP-43 rNLS8 mice can be used as a slower model of disease progression that may be more suitable for long-term therapeutic studies and to monitor early disease mechanisms.

## Materials and Methods

### Animals

All animal procedures were approved by the Macquarie University animal ethics committee (#2022/015) and were performed in accordance with the Australian Code of Practice for the Care and Use of Animals for Scientific Purposes. Transgenic TDP-43 rNLS8 mice for expression of human TDP-43ΔNLS were bred at Australian BioResources (Moss Vale, Australia). Generally, male NEFH-tTA mice (B6: C3-Tg-NEFH-tTA line 8) were crossed with female tetO-hTDP-43ΔNLS mice (B6:C3-Tg-tetO-hTDP-43ΔNLS line 4) to obtain bigenic TDP-43 rNLS8 and monogenic and non-transgenic littermate control mice. The original mice lines were obtained from the Jackson Laboratory (Bar Harbour, ME, USA). Both lines were maintained individually on a mixed B6/C3H background.

### Animal Husbandry

Mice were transferred from ABR and then housed in the central animal facility at Macquarie University at least one week prior to the beginning of experiments to allow for acclimatisation. For all low Dox studies, mice were allocated randomly by personnel independent from the named experimenters to receive either 10 mg/kg or 20 mg/kg in a blinded manner. Experimenters were therefore blinded to treatment. All animals were group housed in individually ventilated cages under identical conditions in a 12-h standard light/dark cycle (22 ± 2 °C, 50–60% humidity), with access to water and food. All mice were fed a chow diet containing 200 mg/kg Dox (Specialty Feeds, WA) until the start of the experiment. h-TDP-43ΔNLS expression was induced at 6–8 weeks of age by switching all mice to a matched standard chow diet lacking Dox for one day (Specialty Feeds, WA), and mice in the Dox off group (no Dox, no DietGel®gel) were maintained on this diet for the duration of the study. For all other groups, the next day, the mice were supplied with a high-energy, nutritionally fortified supplementary diet (DietGel® Boost, ClearH_2_O) to ensure adequate nutrition and hydration as the disease progressed. To minimise variability and avoid sex-specific complications known to occur in the rNLS8 mouse model (Wright et al. 2021), only female mice were used in this study to ensure consistency and reliability in outcome measures, because male mice at 6–8 weeks age are prone to developing urinary retention, which can significantly confound behavioural assessments and survival analyses.

### Doxycycline Administration

Doxycycline (Sigma-Aldrich, D9891) was prepared at concentrations of 10 mg/kg and 20 mg/kg in DietGel®. Mice were supplemented with the Dox-containing DietGel® twice weekly, specifically on Day 1 and Day 3.5 of each week. The mice consumed the entire portion of the DietGel® (4g) during each supplementation. All low-dose Dox supplementation took place between 0900 and 1200 h twice weekly, continuing until either the pre-defined study endpoint or disease end-stage. Disease end-stage was defined as the point at which mice showed severe symptoms requiring humane euthanasia, according to established criteria (Wright et al. 2021). To ensure consistent Dox intake in group-housed animals, we placed Dox-infused DietGel® in multiple feeding stations within each cage to reduce competition and dominance-related feeding behaviour. The average Dox intake per mouse was then estimated by dividing the total amount consumed by the number of mice housed in the cage. Gel consumption was visually monitored daily, and fresh DietGel® was provided only after confirming that the previous portion had been completely consumed. All animals were monitored to ensure that each had consumed the DietGel®. At the endpoint of the study, expression of the human TDP-43 transgene was assessed to confirm that the animals examined had taken in similar levels of Dox.

### Monitoring and Behavioural Assessments

All mice were weighed and assessed in the morning (between 0900 h and 1200 h), once per week as previously described (Walker et al., 2015), from the first day when they were switched to normal diet, until disease end-stage (defined as paralysis of both limbs, slow righting reflex, neurological score of 3 (Wright et al. 2021) and > 20% body weight loss from peak body weight). One week before the diet switch, mice were given two training sessions for both rotarod and grip strength tests.

A digital force meter (Model 47200, Ugo Basile) was used to measure the maximal muscle grip strength from all four limbs to evaluate neuromuscular function and muscle weakness. Mice were held by the tail, placed in the centre of the grid connected to the force meter, and pulled horizontally until  they released the grid. Peak grip strength was recorded in grams, and each mouse was tested three times with a 20-min rest between trials. The highest two readings were averaged for analysis. Grip strength was assessed weekly, except in weeks 2, 3, and 4, when it was assessed twice per week. Disease progression for each mouse was determined as the time in weeks at which grip strength fell below a force of 100 g.

Motor coordination, balance, and endurance were assessed using a rotarod apparatus (Model 7650, Ugo Basile). Mice were placed on a rotating rod, starting at 4 rpm which was gradually accelerated to 40 rpm over 300 s. The time each mouse remained on the rod was recorded, with the longest two trials averaged for analysis. Rotarod testing occurred weekly, except during weeks 2, 3, and 4, when it was assessed twice per week. Disease progression for each mouse  was determined as the time in weeks at which rotarod latency to fall dropped below 50 s.

### Perfusion and Tissue Collection

Mice were deeply anesthetized with a mixture of isoflurane and oxygen and then transcardially perfused with approximately 20 mL of 0.9% sodium chloride solution. Brains and spinal cords were removed and split sagittally at the midline. The left hemisphere was rapidly dissected to isolate the cortex, along with the lumbar spinal cord, which were immediately frozen on dry ice and stored at −80 °C for biochemical analysis. The right hemisphere and remaining tissues were post-fixed in 4% paraformaldehyde (PFA, ProSciTech) in PBS for 24 h at 4 °C, then dehydrated in a sucrose gradient (15–30% sucrose in PBS, Sigma-Aldrich) overnight at 4 °C. Dehydrated tissues were snap-frozen in 2-methyl butane and stored at −80 °C or embedded directly in O.C.T. Compound (Sakura, FineTek), and then cryosectioned at 10 µm thickness on Superfrost Plus slides (Menzel-Glaser) using a Leica CM1950 cryostat for histological analysis.

### Preparation of Tissue Lysates

Brain and spinal cords were thawed on ice and then sonicated in 5 × v/w RIPA buffer (50 mM Tris, 150 mM NaCl, 1% NP-40, 5 mM EDTA, 0.5% sodium deoxycholate, 0.1% SDS, pH 8.0) with phosphatase and protease inhibitors (Sigma). Samples were centrifuged at 4 °C, 100,000g for 30 min and the supernatant taken as the RIPA-soluble fraction. The pellet was washed by sonication with RIPA buffer as above. This supernatant was discarded, and the pellet sonicated in 5 × v/w urea buffer (7 M urea, 2 M thiourea, 4% CHAPS, and 30 mM Tris, pH 8.5) and centrifuged at 22 °C, 100,000g for 30 min. This supernatant was retained as the urea-insoluble fraction. Protein concentrations of the RIPA-soluble fractions were determined using the bicinchoninic acid protein assay (BCA, Pierce).

### Western blotting

Proteins were resolved by SDS-PAGE electrophoresis on (4-20%) BioRad polyacrylamide gels. For all Western blots, 20 μg brain or spinal cord RIPA-soluble protein lysates per sample were added to each lane. Electrophoresis was carried out at 100V for 1h, then proteins were transferred to nitrocellulose membranes using the BioRad Transblot Turbo system. Membranes were incubated with 5% BSA in TBS-T (Tris-buffered saline with  0.1% Tween® 20 detergent ) for 1 h, followed by incubation with primary antibody. Primary antibodies used were mouse anti-human-specific TDP-43 1:5000 (Proteintech Cat# 60019-2-Ig), rabbit anti-phospho-TDP-43 (Ser409/410) 1:1000 (Cosmo Bio Co Cat# CAC-TIP-PTD-P07), and mouse anti-GAPDH 1:,5000 (Proteintech Cat# 60004-1-Ig,) diluted in 5% BSA in TBS-T incubated overnight at 4 °C. Membranes were washed and incubated with anti-mouse/rabbit HRP-conjugated antibodies (1:2000) for 1 h. Immunoreactivity was revealed using the Clarity™ ECL Western Blotting Substrate kit (BioRad) and images were obtained using ChemiDoc MP Imaging System with Image Lab™ software (BioRad). The intensity of each band relative to GAPDH or total protein staining was quantified using ImageJ (v. 1.54f; National Institutes of Health).

### Histological Analysis

Nissl staining was used to identify and quantify the number of α-motor neurons per section in both ventral horn regions of the spinal cord of the mice. Frozen spinal cord sections (10 µm thick) were thawed at room temperature, rinsed in 70% ethanol (Chem-Supply, # EA043-10L-P), rehydrated in distilled water, and stained with 1% Toluidine Blue (Sigma-Aldrich, # T3260). Sections were then washed in distilled water, followed by 70% ethanol, and cover-slipped with DPX mounting medium (Sigma-Aldrich, # 06522). Slides were scanned with a Zeiss Fluorescence Upright AxioImager microscope using a 20 × objective lens. Motor neurons were identified by their large neuronal cell body with intensely blue-stained cytoplasm, reflecting Nissl staining of the rough endoplasmic reticulum. Motor neurons with diameters of 20 µm or greater were specifically counted and classified as α-motor neurons.

### Immunohistochemistry and Confocal Microscopy

Frozen tissue sections were fixed for 15 min in 4% paraformaldehyde (PFA)(Sigma) at room temperature. Slides were then blocked for 1 h in PBS containing 1% BSA (Sigma) and 0.25% (v/v) Triton X-100 (PBST). Primary antibodies, mouse or anti-hTDP-43(Proteintech, #10782-2-AP, 1:1000), or rabbit anti-glial fibrillary acidic protein (GFAP) (Dako, #Z0334, 1:500) were incubated overnight at 4 °C. Secondary goat anti-rabbit Alexa Fluor 488 (Invitrogen, #A11008, 1:2000) was added for 1 h at room temperature. Slides were then incubated for 5 min with 0.5 µg/ml Hoechst 33,258 (Sigma, #94403, 1:500) to stain the nuclei, and mounted onto slides using anti-fade fluorescent mounting media (Dako), leaving  to dry overnight before imaging. Between each step, slides were washed three times in PBST for 3 min each. Confocal microscopy was performed using the Stellaris 5 confocal laser microscope (Leica Biosystems) with a supercontinuum white light laser (400–600 nm). Images were captured at 63 × magnification with multiple Z-stacks taken at 0.3 µm intervals to cover the tissue depth. To avoid bleed-through effects, images at different wavelengths were acquired sequentially in separate tracks. Maximum projection images of the confocal Z-stacks are presented as representative images. For image analysis, Leica Application Suite (LAS) X Office version 1.4.4.26810 (Leica Biosystems) and ImageJ (version 1.54f) software were used. A fixed threshold was applied to each channel based on negative controls (containing secondary antibody only). Image analysis was performed in ImageJ (v. 1.54f; National Institutes of Health).

### Statistical Analyses

Statistical analyses were conducted using GraphPad Prism software, version 8.4.1. Mean differences were evaluated with either one-way ANOVA, two-way ANOVA or unpaired t test, with or without repeated measures as appropriate, or by mixed-effects analysis, followed by Tukey’s multiple comparisons test. Kaplan–Meier survival curves were analysed using the Log-rank (Mantel–Cox) test. Data are presented as mean ± SD or mean ± SEM, and statistical significance was set at *p* < 0.05 for all tests.

## Results

### Low Concentrations of Dox Reduce Soluble TDP-43 ΔNLS Protein Expression and Pathology in TDP-43 rNLS8 Mice

The TDP-43 rNLS8 mice are normally maintained on 200 mg/kg Dox to suppress expression of the transgene, thus disease is initiated when Dox is removed. First, we examined if expression of hTDP-43 ΔNLS could be lowered by retaining Dox supplementation, but using 10–20-fold lower concentrations (10 mg/kg or 20 mg/kg in DietGel® respectively). DietGel® was added to the standard diet two days a week to address specific nutritional needs and to ensure proper hydration and feeding, particularly in this disease model where motor deficits can impair the ability to eat or drink normally. Hence, four groups of mice were created: Dox off (no Dox, no DietGel®), Diet gel only (no Dox + DietGel® ), 10 mg/kg (10 mg/kg Dox + DietGel®), and 20 mg/kg (20 mg/kg Dox + DietGel®). These groups were all monitored from the timepoint when 200 mg/kg Dox was either removed (Dox off and Diet gel only groups) or replaced with 10 mg/kg Dox + DietGel®(10 mg/kg Dox group), or 20 mg/kg Dox + DietGel® (20 mg/kg Dox group). Behavioural phenotypes (grip strength, neurological score, and rotarod performance) were assessed once per week until the Dox off and Diet gel mice reached late disease stage, then assessed weekly until the remaining groups reached end-stage. Body weight was measured once a week, and mice were monitored until disease end-stage. The brain and spinal cords were collected at disease end-stage to examine hTDP-43 ΔNLS expression and pathology. Five disease stages were categorised: pre-onset, disease onset, early disease, late disease stage, and humane end-stage. We applied a stringent humane endpoint for the study of 20% weight loss threshold to prioritise animal welfare in line with institutional guidelines, in contrast to previous studies that have defined 30% weight loss as the humane end-stage criterion (Walker et al., 2015). As detailed below, disease end-stage was reached at 5 and 6 weeks for Dox off and Diet gel mice, and 18 weeks for both 10 mg/kg and 20 mg/kg Dox groups.

**Fig. 1:**
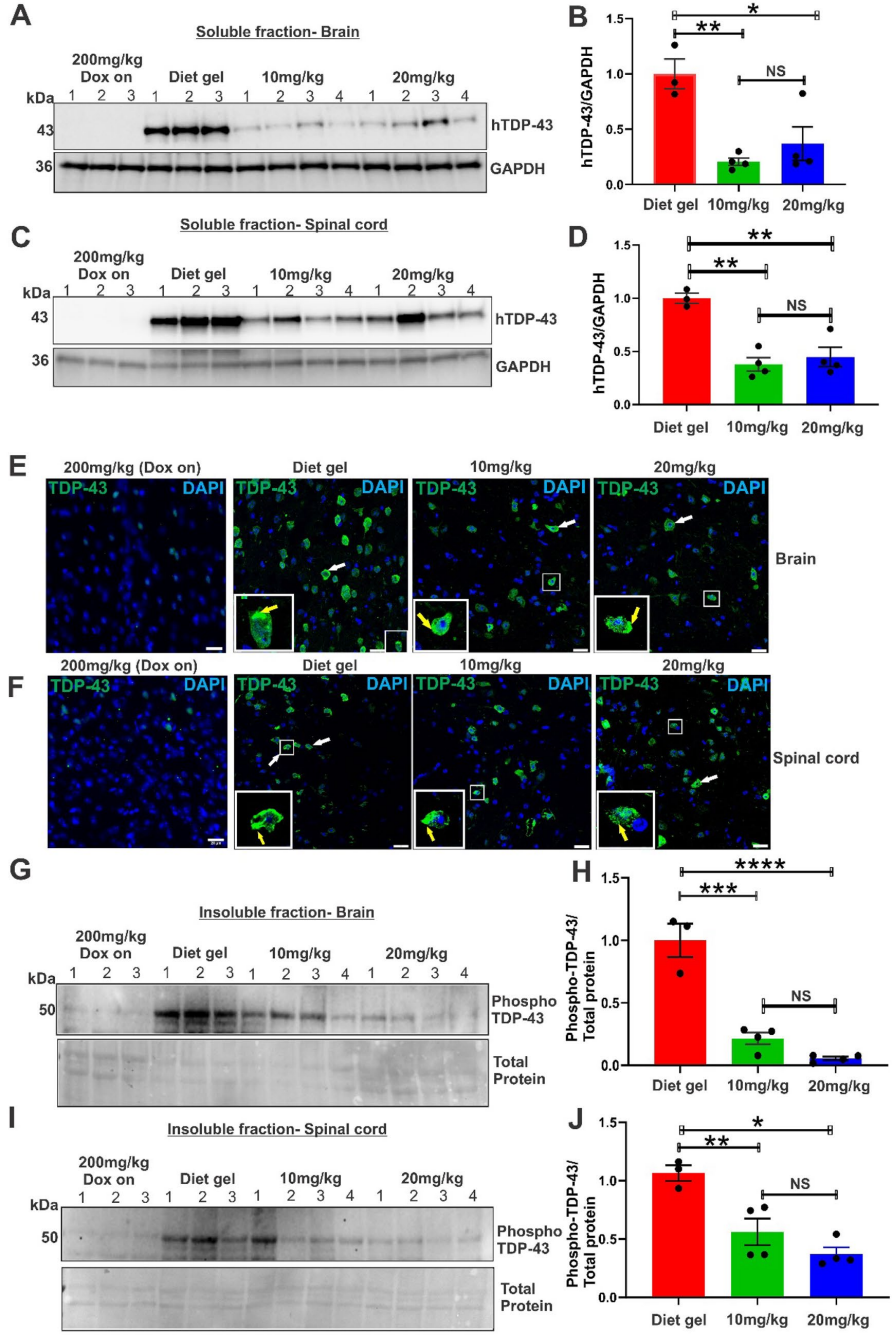
Expression of less phosphorylated, insoluble hTDP-43∆NLS in the brain and spinal cord of TDP-43 rNLS8 mice supplemented with 10 mg/kg and 20 mg/kg Dox. **A** Western blotting using a human-specific TDP-43 antibody (with GAPDH as a loading control) of RIPA-soluble brain lysate fractions prepared from mice analysed in this study: 200 mg/kg Dox on (control), Diet gel (control), 10 mg/kg Dox, and 20 mg/kg Dox. **B** Quantification of Western blots shown in (**A**). Densitometry analysis of relative intensity of h-TDP-43 ΔNLS to GAPDH in brain lysates of Diet gel, 10 mg/kg Dox, 20 mg/kg Dox mice, normalised to the Diet gel group. Data are represented as mean ± SEM, mixed effects analysis, one-way Anova, Tukey’s multiple test comparison, *p < 0.05, **p < 0.01, NS = non-significant (*p* > 0.05), *n*= 3 (Diet gel group), *n* = 4 (10 mg/kg and 20 mg/kg Dox groups). **C** Western blotting using a human-specific TDP-43 antibody (with anti-hTDP-43 and anti-GAPDH as a loading control)  of RIPA-soluble spinal cord lysate fractions prepared from mice analysed in this study: 200 mg/kg Dox on (control), Diet gel (control), 10 mg/kg Dox, and 20 mg/kg Dox. **D** Densitometry analysis of the relative intensity of h-TDP-43 ΔNLS to GAPDH in spinal cord lysates of Diet gel, 10 mg/kg Dox, 20 mg/kg Dox mice, normalised to the Diet gel group. Data are represented as mean ± SEM, mixed effects analysis, one-way Anova, Tukey’s multiple test comparison, **p < 0.01, *n* = 3, NS = non-significant (*p* > 0.05), (Diet gel group), *n* = 4 (10 mg/kg and 20 mg/kg Dox groups). **E**, **F** Representative confocal microscopy maximum projection images following immunohistochemistry using a pan-TDP-43 (detecting both human and mouse TDP-43) antibody (green) and DAPI staining (blue) in the brain (**E**) and spinal cord (**F**) of 200mg/kg Dox on (control), Diet gel alone supplemented mice, 10 mg/kg and 20 mg/kg Dox groups. Scale bar = 20 μM. Cytoplasmic TDP-43 expression is indicated by the white arrows, and *inset* TDP-43 inclusions are indicated by the yellow arrows (**G**) Western blotting using an anti-phospho-TDP-43 (Ser409/410) antibody, and total protein staining in Urea soluble and insoluble brain fractions prepared from Diet gel, 10 mg/kg and 20 mg/kg Dox mice. **H** Quantification of Western blots shown in (**G**). Densitometry analysis of the relative intensity of phospho-TDP-43 (Ser409/410) relative to total protein staining in brain lysates in (**G**). Data are represented as mean ± SEM, mixed effects analysis, one-way Anova, Tukey’s multiple test comparison,***p < 0.001,****p < 0.0001, NS = non-significant (*p* > 0.05), *n* = 3 (Diet gel), *n* = 4 (10 mg/kg and 20 mg/kg Dox groups). **I** Western blotting using an anti-phospho-TDP-43 (Ser409/410) antibody, and total protein staining in Urea soluble and insoluble spinal cord fractions prepared from Diet gel, 10 mg/kg Dox and 20 mg/kg Dox mice. **J** Quantification of Western blots shown in (**I**). Densitometry analysis of the relative intensity of phospho-TDP-43 to total protein staining in spinal cord lysates of mice in (**I**), normalised to the Diet gel group. Data are represented as mean ± SEM, mixed effects analysis, one-way Anova, Tukey’s multiple test comparison, *p < 0.05, **p < 0.01, NS = non-significant (*p* > 0.05), *n* = 3 (Diet gel group), *n* = 4 (10 mg/kg and 20 mg/kg Dox groups). Graphs shown in (**B**), (**D**), (**H**), and (**J**) represent the average of at least two technical replicates of each lysate

Western blotting analyses of whole brain lysates from these animals, and mice where Dox was retained in the diet (Dox on, 200 mg/kg) for comparison, using an antibody specific for human, but not mouse, TDP-43, revealed a statistically significant reduction in soluble h-TDP-43 ΔNLS levels in mice supplemented with either 10 mg/kg Dox (4.8-fold, **p < 0.01) or 20 mg/kg Dox (2.71-fold, *p < 0.05), compared to the Diet gel alone group (Fig. 1A and B). No h-TDP-43 ΔNLS protein was present in the Dox on lysates, demonstrating that 200 mg/kg Dox effectively suppresses its expression at this concentration. No statistically significant differences were detected between the 10 mg/kg and 20 mg/kg Dox groups. Hence, low concentrations of Dox result in significant reductions in h-TDP-43 ΔNLS protein in the mouse brain. Similar Western blotting analyses of whole spinal cord lysates of these animals also revealed significantly less h-TDP-43ΔNLS in mice supplemented with 10 mg/kg Dox (2.64-fold, **p < 0.01) or 20 mg/kg Dox (2.23-fold, **p < 0.01) compared to the Diet gel alone group (Fig. 1C and D). No statistically significant differences were detected between both the 10 mg/kg and 20 mg/kg Dox groups. Hence, 10 mg/kg and 20 mg/kg Dox are enough to express significantly less h-TDP-43 ΔNLS in both the TDP-43 rNLS8 mouse brain and spinal cord compared to the Diet gel only group.

We next examined the presence of TDP-43 pathology in brain and spinal cord neurons of the mice at disease end-stage using immunohistochemistry and confocal imaging. Cytoplasmic h-TDP-43 ΔNLS and cytoplasmic inclusions were detected in both spinal cord and brain tissues in the Diet gel, 10 mg/kg and 20 mg/kg Dox groups (Fig. 1E and F). The insoluble cell pellet fractions (solubilised by urea) were also probed by Western blotting using a phospho-TDP-43 (Ser409/410) specific antibody, representing insoluble, aggregated phosphorylated TDP-43. Significantly less insoluble, phosphorylated h-TDP-43 ΔNLS was present in brains of mice supplemented with either 10 mg/kg or 20 mg/kg Dox (4.65-fold, ***p < 0.001 and 18.8-fold,****p < 0.0001 less**)** compared to the Diet gel only group (Fig. 1G and H). Similarly, significantly less insoluble phosphorylated h-TDP-43ΔNLS was present in spinal cords of mice supplemented with 10 mg/kg or 20 mg/kg Dox (1.78-fold and 2.69 -fold less respectively, **p<0.01 and *p < 0.05) compared to the Diet gel only group (Fig. 1I and J). No statistically significant differences were detected between the 10 mg/kg and 20 mg/kg Dox groups. Together, these results reveal  that both 10 mg/kg and 20 mg/kg Dox supplementation induce expression of TDP-43 with pathological features; cytoplasmic, aggregated, insoluble phosphorylated h-TDP-43 in the mouse brain and spinal cord.

### Low Concentrations of Dox Delay Disease Onset in TDP-43 rNLS8 Mice

To determine whether 10 mg/kg and 20 mg/kg Dox supplementation delay disease onset in TDP-43 rNLS8 mice, analysis of neurological scores was performed weekly. A previously used 4-point scale was used by an observer blinded to the treatment groups (Wright et al., 2021): Score 0: normal, with no observable impairment. Score 1: mild impairment, characterised by abnormal hindlimb splay, slightly slower gait, and a normal righting reflex. Score 2: moderate impairment, indicated by a partially or fully collapsed hindlimb, with at least one foot dragging along the cage floor, and delayed righting reflex. Score 3: severe impairment, with rigid paralysis or minimal movement in the hindlimbs, which can no longer be used for forward motion, and a markedly slow righting reflex (Wright et al., 2021). Disease onset was indicated by the date each animal first reached a neurological score of 1.

**Fig. 2:**
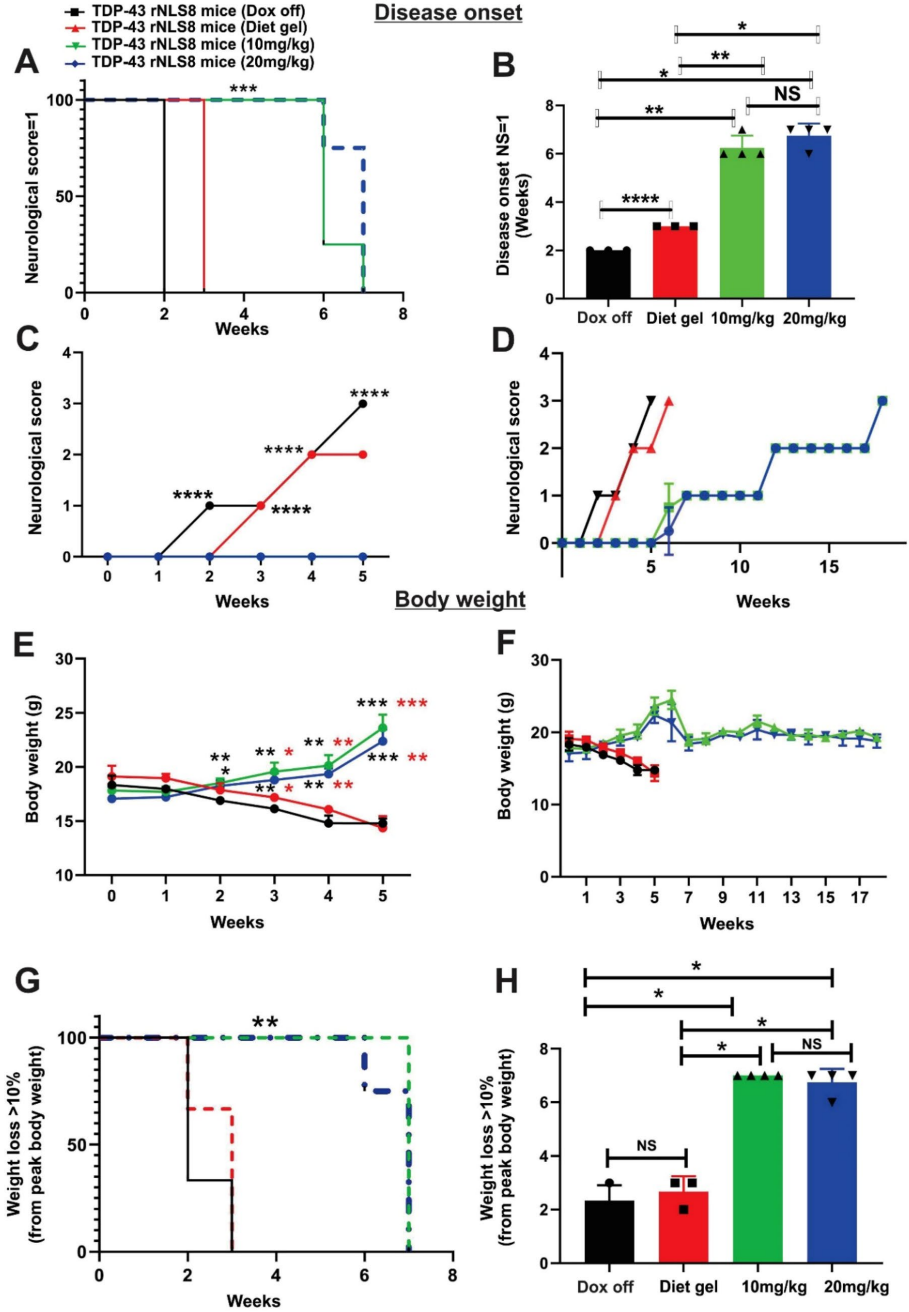
Disease onset is delayed in TDP-43 rNLS8 mice supplemented with 10 mg/kg and 20 mg/kg Dox compared to Diet gel and Dox off groups. **A** Kaplan–Meier curves representing age of disease onset in weeks, defined as an individual mouse achieving a neurological score = 1 (NS = 1). Log-rank (Mantel-Cox) test, ***p < 0.001. Median disease onset; Dox off group = 2 weeks, Diet gel group = 3 weeks, 10 mg/kg Dox group = 6 weeks and 20 mg/kg Dox group = 7 weeks. **B** Disease onset is delayed in 10 mg/kg and 20 mg/kg Dox mice, mean age of onset = 6.25 and 6.75 weeks, respectively, compared to Dox off and Diet gel mice (mean = 2 and 3 weeks, respectively). Mixed effect analysis mean ± SD, one-way Anova, Tukey’s multiple test comparison, ****p < 0.0001, **p < 0.01, *p < 0.05, *n* = 3 (Dox off and Diet gel groups), *n* = 4 (10 mg/kg and 20 mg/kg Dox groups). **C** Neurological scores are significantly higher in both Dox off and Diet gel mice at 2, 3, 4, and 5 weeks compared to 10 mg/kg and 20 mg/kg Dox groups. Mixed effect analysis, two-way Anova, Tukey’s multiple test comparison, mean ± SD, ****p < 0.0001, *n* = 3 (Dox off and Diet gel group), *n* = 4 (10 mg/kg and 20 mg/kg Dox group). **D** Neurological score analysis throughout life until disease end-stage reveals 10 mg/kg and 20 mg/kg Dox mice have delayed disease onset compared to Dox off and Diet gel groups, mean ± SD. **E** Body weights of 10 mg/kg and 20 mg/kg Dox mice are significantly higher than Dox off and Diet gel groups at 2, 3, 4, and 5 weeks, mean ± SD, mixed effect analysis, two-way Anova, Tukey’s multiple test comparison, ***p < 0.001, **p < 0.01, *p < 0.05, *n* = 3 (Dox off and Diet gel group), *n* = 4. Black (*) and red (*) asterisks refer to comparisons to Dox off or Diet gel mice, respectively. **F** Body weight analyses throughout life until disease end-stage reveal 10 mg/kg and 20 mg/kg Dox mice have higher body weight compared to Dox off and Diet gel groups, mean ± SD. **G** Kaplan–Meier curves representing age of disease onset in weeks, defined as an individual mouse achieving a weight loss of > 10% from peak body weight. Log-rank (Mantel-Cox) test, **p < 0.01. Median disease onset; Dox off group = 2 weeks, Diet gel group = 3 weeks, 10 mg/kg Dox group and 20 mg/kg Dox group = 7 weeks. **H** Body weight analyses reveal 10 mg/kg and 20 mg/kg Dox mice (mean = 7 and 6.75 weeks, respectively) have delayed disease onset compared to Dox off and Diet gel groups (mean = 2 and 3 weeks, respectively). Mixed effect analysis of age of disease onset, indicated by age each mouse reaches weight loss of > 10% from peak body weight, mean ± SD, Tukey’s multiple test comparison, *p < 0.05, *n* = 3 (Dox off and Diet gel group), *n* = 4 (10 mg/kg and 20 mg/kg Dox group)

Disease onset was significantly delayed in TDP-43 rNLS8 mice supplemented with 10 mg/kg and 20 mg/kg Dox (median onset = 6 and 7 weeks after removing 200 mg/kg Dox, respectively) compared to both Dox off mice and the Diet gel only group (median onset = 2 and 3 weeks, respectively, all ***p < 0.001) (Fig. 2A). Similarly, the average date of disease onset was significantly delayed in TDP-43 rNLS8 mice supplemented with 10 mg/kg and 20 mg/kg Dox (mean = 6.25, 6.75 weeks after replacing 200 mg/kg Dox, respectively) compared to both Dox off and Diet gel only groups (mean = 2 and 3 weeks, respectively, *p < 0.05 **p < 0.01) (Fig. 2B). A statistically significant difference was detected between the Dox off and Diet gel only supplemented groups (****p < 0.0001), suggesting that the extra nutrition provided by DietGel® delays disease onset by one week. No statistically significant differences in disease onset were obtained between the 10 mg/kg and 20 mg/kg Dox groups (Fig. 2B), despite an apparent visual distinction between the two groups (Fig. 2A). Hence, these data indicate that supplementation with either 10 mg/kg or 20 mg/kg Dox delays disease onset in TDP-43 rNLS8 mice.

We also assessed neurological scores throughout the study until end stage to monitor disease progression. Statistically significant improvements in neurological scores were detected in mice supplemented with 10 mg/kg and 20 mg/kg Dox compared to controls throughout the disease course. Neurological scores in the Dox off group worsened progressively and reached a score of 3 by week 5. In contrast, the Diet gel only group displayed scores of 0 to 2 over the same timeframe, whilst the Dox-supplemented groups (both 10 mg/kg and 20 mg/kg) displayed neurological scores of 0 over the same period (****p < 0.0001, Fig. 2C). Significant improvements in neurological scores in mice supplemented with either 10 mg/kg or 20 mg/kg Dox compared to Dox off and Diet gel only groups throughout the disease course were evident (Fig. 2D).

To confirm these findings, body weights were also analysed weekly to identify disease onset, as previous (Wright et al., 2021). This was determined to be the mean number of weeks after replacing 200 mg/kg Dox until the loss of 10% weight was reached from peak body weight (Wright et al., 2021). A statistically significant difference in body weights was detected from week 2 onwards in TDP-43 rNLS8 mice supplemented with either 10 mg/kg or 20 mg/kg Dox compared to both Dox off and Diet gel supplemented groups (***p < 0.001,**p < 0.01, *p < 0.05 weeks) (Fig. 2E). The 10 mg/kg and 20 mg/kg Dox mice initially gained weight but then began to lose weight at approximately 6 weeks (Fig. 2F).

Disease onset was significantly delayed in TDP-43 rNLS8 mice supplemented with 10 mg/kg and 20 mg/kg Dox (median = 7 weeks for both groups) compared to both Dox off and Diet gel only groups (median = 2 and 3 weeks, respectively, all **p < 0.01) (Fig. 2G). Similarly, the average date of disease onset was significantly delayed in TDP-43 rNLS8 mice supplemented with 10 mg/kg or 20 mg/kg Dox (mean = 7 and 6.75 weeks, respectively) compared to both Dox off and Diet gel only groups (mean = 2 and 3 weeks, respectively, both *p < 0.05 (Fig. 2H). No statistically significant differences were detected between the Dox off and Diet gel groups, nor between the 10 mg/kg and 20 mg/kg Dox groups, the latter implying that 10 mg/kg is sufficient to delay disease onset in TDP-43 rNLS8 mice. These findings suggest that 10 mg/kg and 20 mg/kg Dox supplementation delays disease onset in TDP-43 rNLS8 mice by at least 4 weeks.

### Low Concentration of Dox Delays Disease Progression in TDP-43 rNLS8 Mice

To determine whether 10 mg/kg and 20 mg/kg Dox supplementation delays onset of motor dysfunction and disease progression in TDP-43 rNLS8 mice, we examined behavioural phenotypes previously described in this model throughout the disease course. First, the accelerating rotarod test was used to monitor motor coordination, balance, and endurance. Disease progression, from the early disease stage to the late disease stage, by measuring the latency to fall from the rotarod with a threshold of over 50 s was also assessed.

**Fig. 3:**
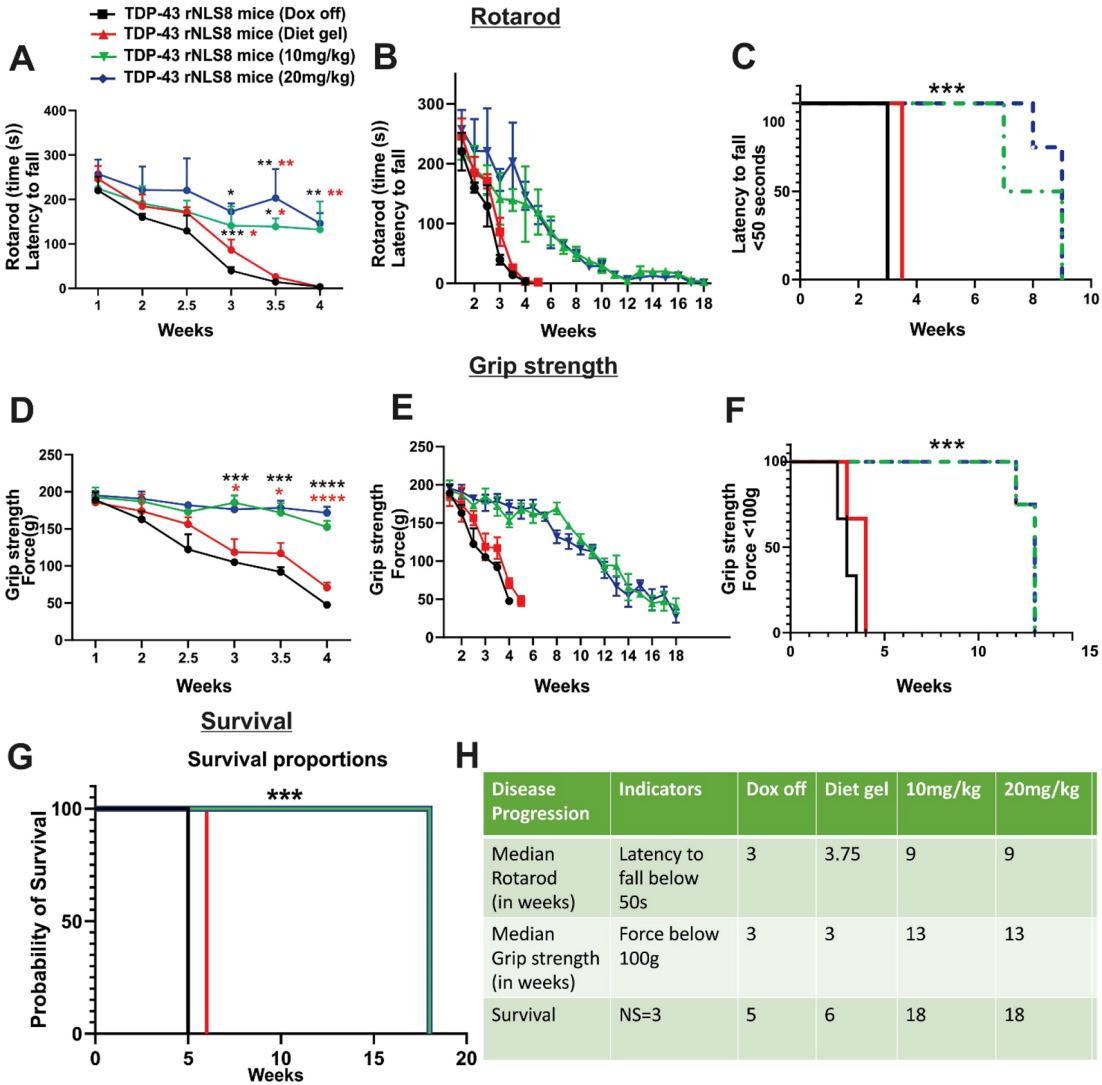
Motor performance, grip strength, and survival are improved in TDP-43 rNLS8 mice supplemented with 10 mg/kg and 20 mg/kg Dox compared to Diet gel and Dox off groups. **A** Rotarod performance (latency to fall in seconds) is significantly improved at 3, 3.5, and 4 weeks in 10 mg/kg and 20 mg/kg Dox mice compared to Dox off and Diet gel  groups, indicating improvement in motor function. Mixed effects analysis, mean ± SD, two-way Anova, Tukey’s multiple test comparison, ***p < 0.001, **p < 0.01, *p < 0.05, *n* = 3 (Dox off and Diet gel groups), *n* = 4. (10 mg/kg and 20 mg/kg Dox groups). Black (*) and red (*) asterisks refer to comparisons to Dox off or Diet gel mice respectively. **B** Rotarod performance (latency to fall in seconds) throughout life until disease end-stage reveals the improved rotarod performance of 10 mg/kg and 20 mg/kg Dox mice compared to Dox off and Diet gel mice, mean ± SD. **C** Kaplan–Meier curves representing rotarod latency to fall, defined as an individual mouse < 50s. Severe motor deficits were detected by poor performance on the rotarod test, with latencies to fall dropping below 50 s. Log-rank (Mantel-Cox) test, ***p < 0.001. 10 mg/kg and 20 mg/kg Dox mice display improved performance compared to Dox off and Diet gel groups, median = 9 weeks for both, in contrast to 3 and 3.5 weeks for Dox off and Diet gel groups, respectively. **D** Muscle function, indicated by grip strength, is significantly improved at 3, 3.5, and 4 weeks in 10 mg/kg and 20 mg/kg Dox mice compared to Dox off and Diet gel mice. Mixed effects analysis, mean ± SD, two-way Anova, Tukey’s multiple test comparison, ****p < 0.0001, ***p < 0.001, *p < 0.05, *n* = 3 (Dox off and Diet gel group), *n* = 4 (10 mg/kg and 20 mg/kg Dox group). Black (*) and red (*) asterisks refer to comparisons to Dox off or Diet gel mice respectively. **E** Muscle function, indicated by grip strength, throughout life until disease end-stage reveals improved grip strength of mice supplemented with 10 mg/kg and 20 mg/kg Dox compared to both Dox off and Diet gel groups, mean ± SD. **F** Kaplan–Meier curves representing grip strength, defined as an individual mouse achieving < 100(g) force. Log-rank (Mantel-Cox) test, ***p < 0.001. 10 mg/kg and 20 mg/kg Dox display improved muscle function compared to Dox off and Diet gel groups, median = 13 weeks for both compared to 3 for Dox off and Diet gel groups. Severe motor deficits were  detected by poor muscle function, with grip strength dropping below 100 g force. **G** Kaplan–Meier curves representing probability of survival, defined as an individual mouse achieving the humane endpoint criterion. Log-rank (Mantel-Cox) test, ***p < 0.001. 10 mg/kg and 20 mg/kg Dox mice reach a neurological score of 3 at a median of 18 weeks compared to 5 or 6 weeks for the Dox off or Diet gel groups, respectively. **H** Table summarising disease progression in all groups, as assessed by grip strength, rotarod performance, and survival

Consistent with previous observations, a dramatic decline in motor performance was evident in the Dox off and Diet gel alone groups, at 3, 3.5, and 4 weeks after Dox removal, respectively. Rotarod performance was significantly improved in mice supplemented with 10 mg/kg and 20 mg/kg Dox from week 3 onwards (weeks 3, 3.5, and 4 weeks (***p < 0.001, **p < 0.01, *p < 0.05)), until the Dox off group and Diet gel alone groups reached late stage (Fig. 3A and B). There were no significant differences between 10 mg/kg and 20 mg/kg for the full duration of the experiment, demonstrating there was no effect on increasing the concentration of Dox from 10 to 20 mg/kg on rotarod performance (Fig. 3A and B). Disease progression from onset to early and late disease stage was assessed by the latency to fall from the rotarod below 50 s, which was significantly delayed in TDP-43 rNLS8 mice supplemented with 10 mg/kg and 20 mg/kg Dox (median = 9 weeks after replacement of 200 mg/kg Dox, respectively) compared to both Dox off mice and Diet gel only group (median 3 and 3.75 weeks, respectively, all ***p < 0.001) (Fig. 3C).

Next, grip strength of the mice was analyzed to monitor changes in muscle strength, both in the forelimbs and hindlimbs. Consistent with previous observations (Walker et al., 2015; Wright et al., 2021), dramatic muscle weakening was detected from 3 weeks onwards in the Dox off group, and in the Diet gel alone group. Grip strength was significantly greater in mice supplemented with 10 mg/kg and 20 mg/kg Dox from week 3 onwards (weeks 3, 3.5 and 4 weeks, ****p < 0.0001, ***p < 0.001, *p < 0.05) (Fig. 3D and E) until the disease late stage was reached by the Dox off and Diet gel groups**.** There were no differences between 10 mg/kg and 20 mg/kg for the full duration of the experiment, demonstrating no effect of increasing the concentration of Dox from 10 to 20 mg/kg (Fig. 3D and E). Disease progression from onset to early and late disease stage, measured by muscle strength of an individual mouse reaching a force below 100 g, was significantly delayed in TDP-43 rNLS8 mice supplemented with 10 mg/kg or 20 mg/kg Dox (median = 13 weeks, respectively), compared to both Dox off mice and Diet gel only groups (median = 3 and 4 weeks, respectively, all ***p < 0.001) (Fig. 3F). Hence, low Dox supplementation delays disease progression and the onset of motor deficits.

### Low Concentrations of Dox Extend Survival in TDP-43 rNLS8 Mice

Next, we examined whether low Dox supplementation extends lifespan in the TDP-43 rNLS8 mouse model. The humane endpoint was identified by the age at which the mice reached a neurological score =3 (complete paralysis of both limbs, slow righting reflex and > 20% body weight loss). Kaplan–Meier analysis revealed a statistically significant increase in survival in TDP-43 rNLS8 mice supplemented with 10 mg/kg or 20 mg/kg Dox (median = 18 weeks each) compared to both Dox off mice and Diet gel only groups (median = 5 and 6 weeks, respectively) (Fig. 3G and H; χ^2^ = 18.46, df = 3 ***p < 0.001). Previously, survival in the TDP-43 rNLS8 mouse was reported to be 10 weeks following Dox removal using 30% weight loss as the end-stage criterion (Walker et al., 2015). However, our findings using the more stringent 20% weight loss threshold-showing that the Dox off mice survive only to 5–6 weeks following Dox removal-are consistent with other studies within our laboratory (data not shown). Hence overall, these results indicate that low concentrations of Dox effectively slow the impairment of motor deficits and grip strength, and extend survival in the TDP-43 rNLS8 mouse model.

### Loss of Motor Neuron and Neuroinflammation are Present in Low Dox TDP-43 rNLS8 Mice at Disease end-Stage

**Fig. 4:**
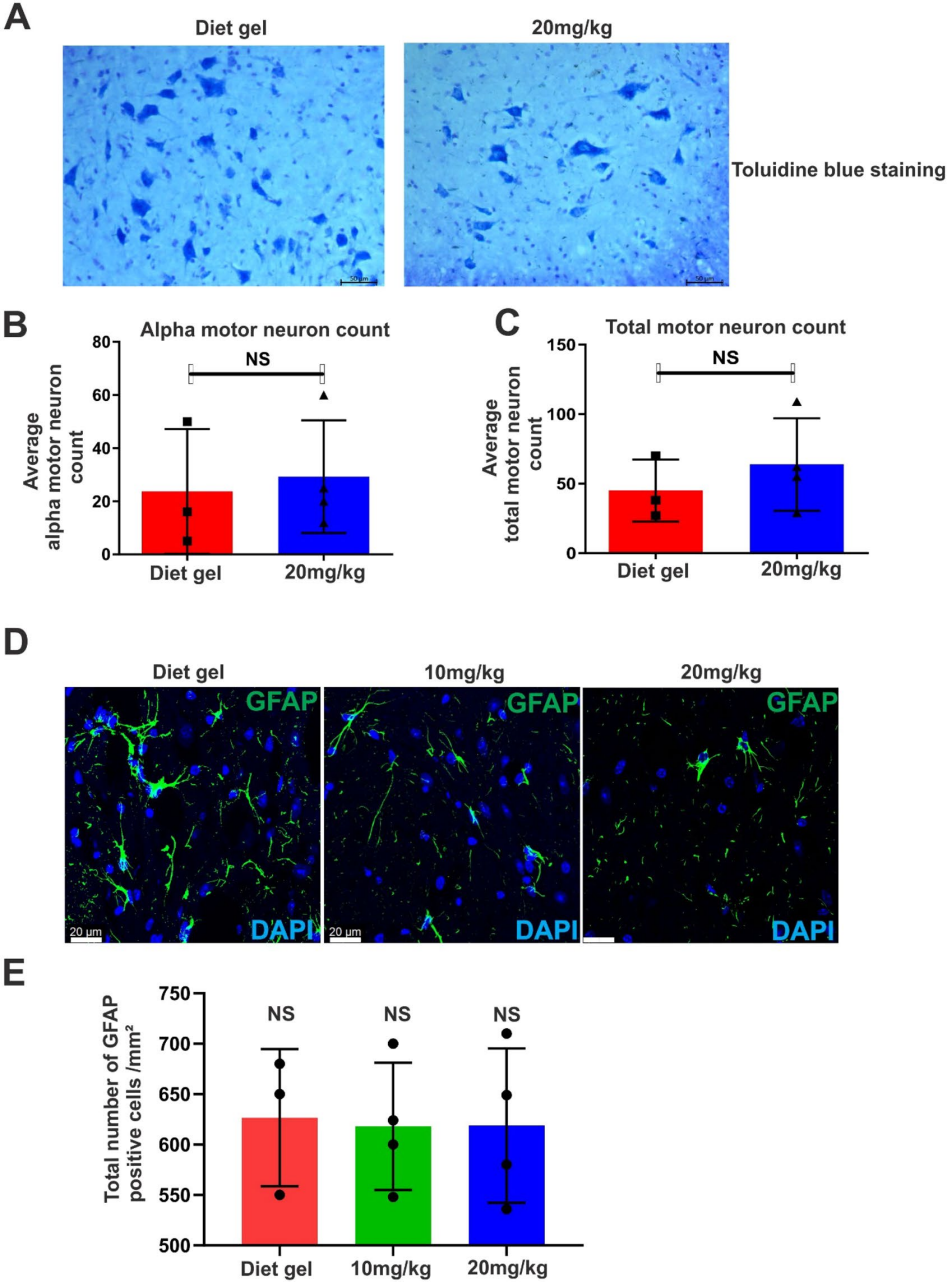
Loss of motor neurons and neuroinflammation are present in low Dox TDP-43 rNLS8 mice at disease end-stage. **A** Bright-field microscopy following Nissl staining using Toluidine blue of both ventral horn regions of the lumbar spinal cord (section thickness: 10 µm) from Diet gel and 20 mg/kg Dox mice, scale bar = 50 μm. **B** Quantification of images in (**A**) reveals there were no differences in the total number of α-motor neurons, identified by Nissl-stained cells with a diameter > 20 μM, present in Diet gel and 20 mg/kg Dox mice at disease end-stage (six weeks and 18 weeks, respectively)   two-tailed, unpaired *t* test, NS = non-significant (*p* > 0.05). **C** Quantification of images in (**A**) reveals there were no differences in total number of motor neurons (both α and γ), identified by the presence of all cells with large soma and intense Nissl-staining, present in Diet gel and 20 mg/kg Dox mice at disease end-stage (six weeks and 18 weeks respectively), two-tailed, unpaired *t* test, NS = non-significant (*p* > 0.05). **D** Representative confocal microscopy maximum projection images following immunohistochemistry of lumbar spinal cord sections (10 µm thickness) using anti-GFAP antibodies (green), co-stained with nuclear marker DAPI (blue) of Diet gel, 10 mg/kg Dox, and 20 mg/kg Dox mice, scale bar = 20 μm. **E** Quantification of images in (**D**) reveals there were no differences in the total number of GFAP-positive cells/mm^2^ between groups. Mixed effects analysis mean ± SD, one-way Anova, Tukey’s multiple test comparison, NS = non-significant (*p* > 0.05), *n*= 3 (Diet gel group), *n* = 4 (10 mg/kg and 20 mg/kg Dox group)

We then examined tissues from disease end-stage animals to determine if the same pathological characteristics are present in mice treated with 10 mg/kg or 20 mg/kg Dox . Neuroinflammation and motor neuron degeneration are known features present in the TDP-43 rNLS8 model (Spiller et al., 2016; Walker et al., 2015). Motor neuron loss begins two weeks after Dox removal, with significant loss by the sixth week, particularly in the spinal cord, when approximately 30% of motor neurons are lost. We first examined motor neuron loss in mice supplemented with 20 mg/kg Dox at disease end-stage. The total number of all motor neurons (both α and γ), and α-motor neurons only, remaining in  the ventral horn regions of the spinal cord following Nissl staining was quantified as previously described (Huang et al., 2012; Mitchell et al., 2015). Motor neurons were identified by the presence of cells with large soma and intense blue cytoplasmic Nissl staining, whereas α-motor neurons were specifically detected by the presence of Nissl-stained cells with diameters of > 20 μm (C. Huang et al., 2012; Mitchell et al., 2015). However, no significant differences in the numbers of either total or α-motor neurons were detected between the Diet gel and the 20 mg/kg Dox groups at end-stage (Fig. 4A–C). Neuroinflammation was then assessed by GFAP immunohistochemistry of  brain tissues of TDP-43 rNLS8 mice. However, no significant differences in the total number of GFAP-positive cells were detected between the Diet gel, 10 mg/kg or 20 mg/kg Dox groups (Fig. 4D–E) at disease end-stage. Hence, despite the slowed disease course and extension of lifespan by low Dox supplementation, two pathological features—motor neuron loss and neuroinflammation—are retained in the TDP-43 rNLS8 mice once animals have reached disease end-stage.

As no significant differences were detected between the 10 mg/kg and 20 mg/kg groups for any of the parameters assessed, the data from these two treatment groups were combined to increase the statistical power of the analysis. Again, a statistically significant delay in disease onset was present compared to Dox off and Diet gel only groups, detected by either neurological score (Supplementary Fig. 2A, ****p < 0.0001) or body weight loss (Supplementary Fig. 2B, ***p < 0.001). Similarly, a statistically significant delay in motor performance, grip strength or lifespan was present compared to Dox off and Diet gel only groups, (Supplementary Fig. 3A, *p < 0.05, ***p < 0.001, Supplementary Fig. 3B,,****p < 0.0001, Supplementary Fig. 3C, ****p < 0.0001, *p < 0.05, Supplementary Fig. 3D ***p < 0.001, Supplementary Fig. 3E, ****p < 0.0001). We also performed post-hoc power calculations of the separate 10 mg/kg and 20 mg/kg groups using the observed effect sizes and variability, which revealed the study was sufficiently powered (power > 95%) to detect statistically significant differences at *α*<0.05 when the 10 mg/kg and 20 mg/kg groups were analysed separately. This suggests that the magnitude of the treatment effect was large enough to be reliably detected with the initial sample size.

## Discussion

The TDP-43 rNLS8 model offers significant improvements over previous TDP-43 in vivo models in replicating the combined core features of ALS/FTD pathology: motor neuron loss, muscle denervation/atrophy, and a progressive motor phenotype with premature death (Walker et al., 2015). This highlights the relevance of this mouse to study disease pathogenesis. However, the NEFH promoter drives higher levels of expression in this model compared to the previously used Camk2a promoter: tenfold vs eightfold in the cortex, respectively (Igaz et al., 2011; Walker et al., 2015). Overexpression models of TDP-43 have been recently criticised in ALS/FTD (Carmen-Orozco et al., 2024) because high levels of TDP-43 expression in mice result in skipping of constitutive exons that are typically included in mRNAs under normal conditions (Carmen-Orozco et al., 2023; Carmen-Orozco et al., 2024). Hence, high levels of overexpression in TDP-43 mice models may not truly reflect human disease (Carmen-Orozco et al., 2023; Carmen-Orozco et al., 2024). Here, we predicted that lower levels of hTDP-43 ΔNLS expression would result in more physiologically relevant levels of TDP-43, whilst retaining the relevant pathological and behavioural features. This was achieved using low levels of Dox supplementation to slow disease progression  and thus more closely replicate human disease. The findings of this study suggest that this approach provides a useful and novel platform for studying ALS/FTD pathogenesis and testing potential therapies.

We demonstrate that reducing the concentration of Dox in the diet from 200 mg/kg to 10 or 20 mg/kg lowers hTDP-43 ΔNLS protein expression significantly, by 2.7–4.8-fold in the brain and by 2.23–2.6-fold in the spinal cord. Lower levels of phosphorylated TDP-43 (409/410), as a marker of TDP-43 pathology, were also present in the brain (4.65–18.8-fold) and spinal cord (1.78–2.69-fold). Consequently, this delays disease onset ~ two–threefold, as assessed by both loss of body weight (from peak body weight) and neurological scores, from a mean/median onset of ~ 6–7 weeks in both 10 mg/kg and 20 mg/kg Dox groups, compared to Dox off mice and Diet gel groups, with a mean/median onset of 2 and 3 weeks, respectively. Disease progression was then delayed by ~ two–fourfold, as assessed by both accelerating rotarod performance and grip strength. The median decline in rotarod performance was reached at 9 weeks in the 10 mg/kg and 20 mg/kg Dox groups compared to the Dox off and Diet gel groups, where the medians were 3 and 3.75 weeks, respectively. Similarly, the median onset of muscle weakness, as determined by grip strength, was reached in 10 mg/kg and 20 mg/kg Dox- supplemented mice at 13 weeks, compared to the Dox off and Diet gel groups, where the medians were 3 and 4 weeks, respectively. This demonstrates that using this approach, low concentrations of Dox reduce hTDP-43 ΔNLS expression, leading to delayed behavioural phenotypes and survival. However, hTDP-43 ΔNLS is still expressed in motor neurons in both the brain and spinal cord, where the expected pathological features are present. This indicates that the low Dox approach  effectively mirrors the functional impact of TDP-43 pathology and associated behavioural phenotypes, providing a valuable tool for studying disease progression. Therefore, low levels of Dox (10 mg/kg and 20 mg/kg) enable a more gradual disease onset and extension of disease course, thus more closely mimicking the progression of ALS in humans.

**Fig. 5:**
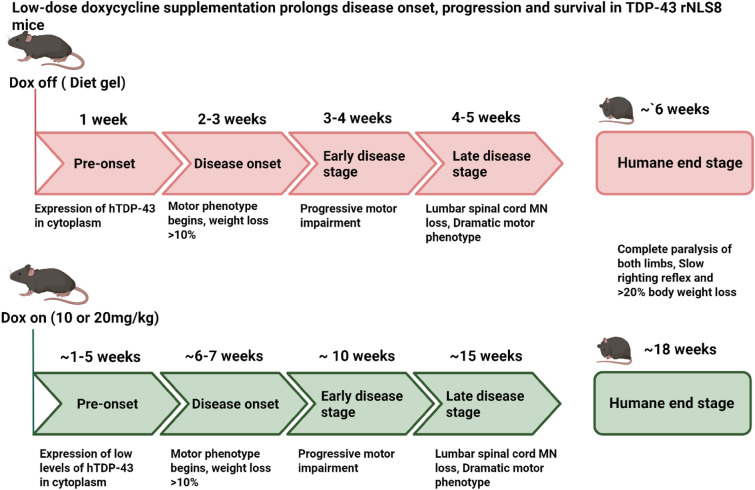
Low-dose Dox supplementation prolongs disease onset, progression, and survival in TDP-43 rNL8 mice. The schematic diagram illustrates a comparative timeline showing key stages in disease progression between the Dox off TDP-43 rNLS mouse model as assessed here and the modified approach described in this study, using low-dose doxycycline (Dox, 10-20 mg/kg) added to the diet. *Pre-symptom onset* When Dox (200 mg/kg) is removed completely (Dox off and Diet gel groups), *top panel*), the pre-symptom onset stage lasts for one week, whereas when 200 mg/kg Dox is replaced with lower concentrations (Dox on [10 or 20 mg/kg], *bottom panel*), this stage continues for five weeks. *Disease onset* marked by onset of motor symptoms, follows at two-three weeks in Dox off and Diet gel mice, but this is delayed until six weeks for 10 mg/kg animals, or seven weeks for 20 mg/kg low-dose Dox-supplemented mice. *Early disease stages* This pattern of symptom delay continues, and the early disease stage, which is marked by continued progressive motor impairment, is reached at three-four  weeks in Diet gel mice, but this is  extended to ten weeks in both groups of low-dose Dox-supplemented animals. *Late disease stage* is defined as severe motor dysfunction, which is reached at four-five weeks in Diet gel mice, but this is not attained until fifteen weeks in the low-dose Dox animals. *Humane end-stage* is reached at six weeks in Dox off mice (using > 20% body weight loss) but this is extended to eighteen weeks in the low-dose Dox animals. Hence using the low-dose Dox approach, disease onset and progression are slowed, and survival is enhanced in TDP-43 rNL8 mice. This offers an alternative strategy to examine early disease mechanisms and perform preclinical therapeutic testing using this ALS mouse model

Using this approach, by administering low doses of Dox, each disease stage was significantly extended (Fig. 5). In the Dox off and  Diet gel mice, the pre-onset stage begins at 1 week off Dox, whereas using 10 mg/kg or 20 mg/kg Dox, this is extended up to 5 weeks following replacement of 200 mg/kg Dox. The disease onset stage follows at 2–3 weeks in the Dox off and Diet gel mice, contrasting with a slower onset at 6–7 weeks using  low-dose Dox supplementation. This pattern of delay continues through the subsequent stages. The early disease stage appears at 4 weeks in the Dox off and Diet gel mice, but this does not begin until 10 weeks in the 10 mg/kg or 20 mg/kg Dox treated animals. The late disease stage begins at 5 weeks in the Dox off and Diet gel mice, whereas this stage is reached at 15 weeks following replacement of 200 mg/kg Dox in the low-dose Dox treated mice (Fig. 5). Survival was also increased by three-fold using 10 mg/kg or 20 mg/kg Dox from 6 weeks to 18 weeks using the more stringent < 20% body weight loss criteria. These results therefore reveal the potential of fine-tuning Dox levels to optimise the balance between disease progression and functional outcomes, thereby improving the utility of TDP-43 rNLS8 mice in preclinical research. The slower decline in disease and behavioural phenotypes, and the extended timelines using this approach thus provide a framework for assessing early disease mechanisms in ALS or examining new treatments preclinically (Fig. 5). However, it is important to note that at disease end-stage, there were no significant differences between the 20 mg/kg Dox and Diet gel mice, implying that the same endpoint is reached, although progression is slowed.

Several therapeutic interventions have been tested using the TDP-43 rNLS8 model, but few have yielded successful outcomes. Riluzole did not improve disease onset, atrophy of hindlimb muscle, or TDP-43 pathology, and it had no effect on progression of late-stage disease or survival (Wright et al., 2021). Similarly, ASO-mediated knockdown of the integrated stress response mediator *Chop* had little effect on motor deficits and did not ameliorate TDP-43 pathology in this model (Luan et al., 2023). Reduction of matrix metalloproteinase 9 (MMP-9) protected motor neurons but led to premature death in a subset of TDP-43 rNLS8 mice (Spiller et al., 2019). Monoclonal antibodies against the glycine-rich domain of TDP-43 had no effect on body weight loss, phospho-TDP-43 levels, or neuroinflammation (Riemenschneider et al., 2023), although neurofilament levels were significantly reduced in treated TDP-43 rNLS8 animals (Riemenschneider et al., 2023). Similarly, suppressing miR-23a production in TDP-43 rNLS8 mice accelerated disease progression, as measured by motor function (Tsitkanou et al., 2022). The challenges faced in these studies may relate to the rapid disease course produced by high levels of overexpression of hTDP-43 ΔNLS in this model, creating a short therapeutic window. However, a recent study reported improved lifespan and motor phenotype and decreased neuroinflammation following intracerebroventricular injection of AAV9/NF242 in this model, demonstrating that in some instances it can be used (Droppelmann et al., 2024).

There are several limitations of this study. Whilst a small sample size was used, statistical significance was nevertheless achieved in the disease parameters assessed. Furthermore, as no statistically significant differences were detected between 10 and 20 mg/kg Dox, we combined these data together to increase the statistical power of the study (Supplementary Figs. 2 and 3). In addition post-hoc power analyses revealed that the study was sufficiently powered (> 95%) with separate 10 mg/kg and 20 mg/kg groups to detect statistically significant differences at α<0.05. Hence the magnitude of the treatment effect was large enough to be reliably detected with the initial sample size. Second, we examined TDP-43 pathology, protein expression, and neuroinflammation at the disease end-stage only. In the future, it would be worthwhile to examine these features at early disease stages in 10 and 20 mg/kg Dox-supplemented mice. We also acknowledge that the use of group housing in this study can introduce limitations regarding the uniform intake of Dox in these animals. Social hierarchy, individual feeding behaviours, and metabolic differences among mice can lead to variability in consumption, which may, in turn, affect the consistency of transgene activation in inducible models. However, we opted for group housing to comply with animal welfare guidelines and to minimise stress, which can otherwise confound behavioural and physiological measures. To mitigate this limitation, as detailed in the Material and Methods, we also ensured regular monitoring of food intake at the cage level and maintained consistent group sizes. Importantly, similar levels of hTDP-43 transgene were expressed in the mice examined in this study. Hence, we believe that our approach represents a balanced compromise between experimental control and animal wellbeing. Nevertheless, despite these limitations, the findings of this study are important in identifying new approaches to examine disease onset, progression, pathology, and survival more thoroughly in this model. Further refinement of the TDP-43 rNLS8 model through the careful modulation of Dox concentration is thus a highly promising intervention that warrants further attention.

The low Dox TDP-43 rNLS8 mice can also be used in conjunction with other TDP-43 ALS mouse models to provide unique insights into pathophysiology. Transgenic TDP-43^Q331K^, TDP-43^A315T^, and TDP-43^N390D^ mice each capture distinct aspects of ALS and FTLD pathology, thus overall enhancing our understanding of these complex diseases. The TDP-43^Q331K^ model is characterised by a progressive decline in muscle mass and motor function, with a 43% decrease in hindlimb muscle mass by 6 months and a 73% reduction in muscle action potential by 8 months (Watkins et al., 2021). Hence, this model is useful for studies examining motor function and testing interventions aimed at muscle preservation (Watkins et al., 2021). TDP-43 pathology appears but at a much later timepoint than TDP-43 rNLS8, at 24 months of age. The low Dox supplementation TDP-43 rNLS8 strategy also results in similar phenotypes as this model, but with a much earlier appearance of TDP-43 pathology. In contrast, the TDP-43^A315T^ model exhibits an aggressive disease course in males, with rapid onset and mortality before 15 weeks, whereas females show a delayed progression, surviving up to 20–35 weeks (Mascho et al., 2024). This model presents severe respiratory impairments, loss of hypoglossal and phrenic motor neurons, and increased glial activation. Thus, it is particularly suitable for studying respiratory failure and testing respiratory-targeted therapies in ALS (Mascho et al., 2024). The TDP-43^N390D^ CRISPR knock-in model displays male-dominant age-dependent, pathological, and behavioural characteristics of ALS (S.L. Huang et al., 2020), but with a much longer disease course in males compared to females (average survival of 25.5 ± 6 months) (S.-L. Huang et al., 2020). These models highlight the varying pathogenic pathways linked to TDP-43 mutations, allowing researchers to explore muscle, respiratory, and broad ALS pathologies (White et al., 2018).

In conclusion, using low concentrations of Dox represents a promising strategy for improving the fidelity of the TDP-43 rNLS8 model in recapitulating human ALS/FTD pathology with a delayed disease onset and progression. This approach may provide valuable insights into early-stage disease mechanisms and offers an alternative platform to test effective targeted therapies in this model.

## Supplementary Information

Below is the link to the electronic supplementary material.

Supplementary file1 (DOCX 729 KB)

## Data Availability

The data supporting the results of this study are available upon reasonable request to the corresponding author.
