# Supplementary material for: A Slower-Progressing TDP-43 rNLS8 Mouse Model for ALS: Implications for Preclinical and Mechanistic Studies
Source: Neuromolecular Med. 2025 Aug 18;27(1):59. doi: 10.1007/s12017-025-08871-z (PMC12361281; doi:10.1007/s12017-025-08871-z)
Supplement: Supplementary file 1 — Supplementary file1 (DOCX 729 KB) [file 12017_2025_8871_MOESM1_ESM.docx]

**A Slower-Progressing TDP-43 rNLS8 Mouse Model for ALS: Implications for Preclinical and Mechanistic Studies**

Cyril Jones Jagaraj^1^, Prachi Mehta^1^, Julie Hunter^1^, Julie D Atkin^1,2^

**Author affiliations:**

^1^ MND Research Centre, Macquarie Medical School, Faculty of Medicine, Health and Human Sciences, Macquarie University, Sydney, NSW 2109, Australia.

^2^ La Trobe University, Bundoora, Melbourne, VIC 3086, Australia.

Correspondence to

Prof. Julie D Atkin

Macquarie University

75 Talavera Road NSW 2109

Phone: 02 9850 2772

Email ID: julie.atkin@mq.edu.au

**Running title**: Extending disease in TDP-43 rNLS8 ALS mice

**Keywords:** ALS/MND, TDP-43 pathology, Longer disease course

**Supplementary Figure 1**


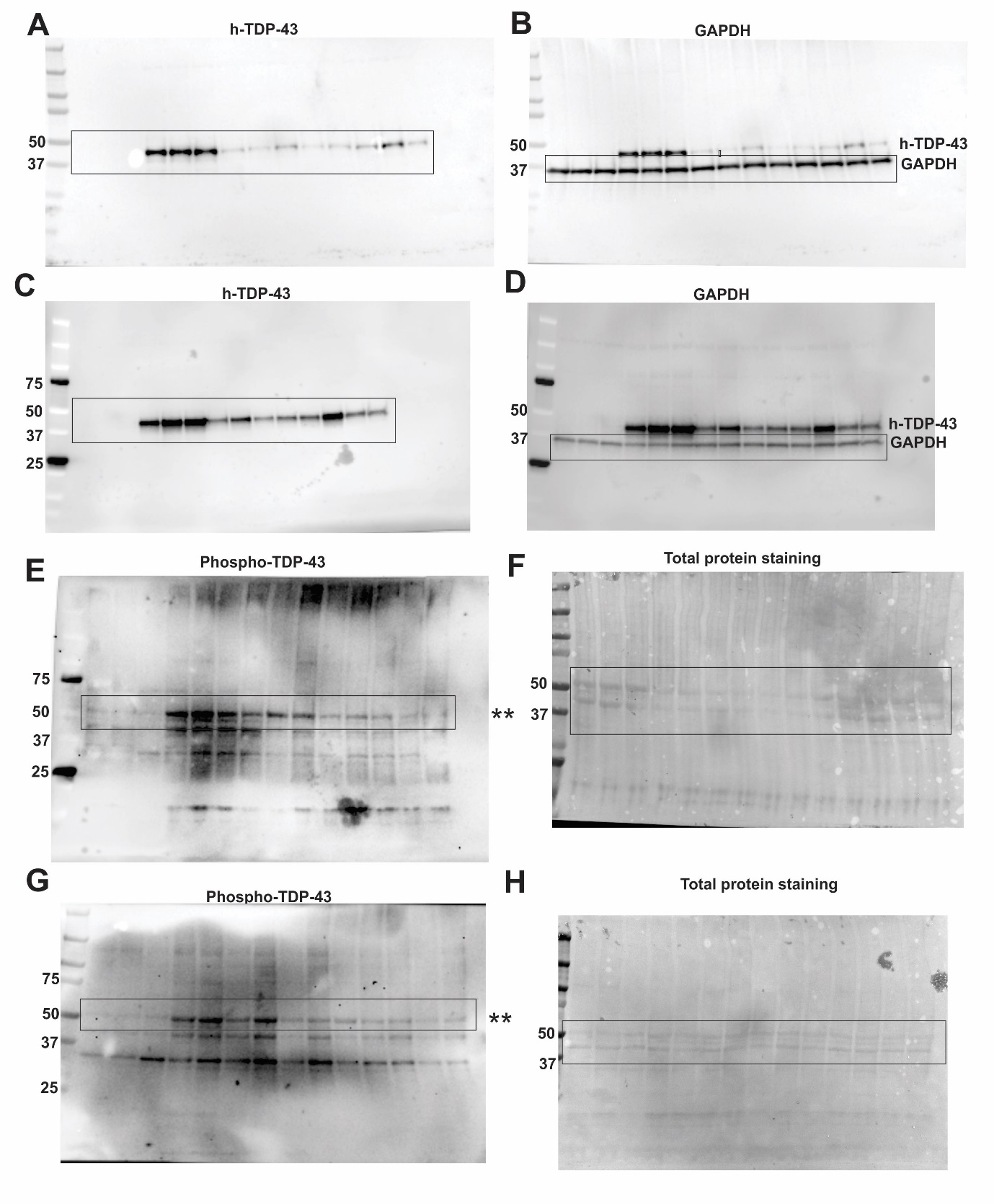


**(A and B)** Full-length Western blots shown in Figure 1A

**(C and D)** Full-length Western blots shown in Figure 1C

**(E and F)** Full-length Western blots shown in Figure 1G

**(G and H)** Full-length Western blots shown in Figure 1I

**(A-H)** boxes indicate region of gel shown in each figure.

**Supplementary Figure 2**

**Combined analysis of disease onset of 10mg/kg and 20mg/kg Dox mice and controls (Dox off and Diet gel).**

**
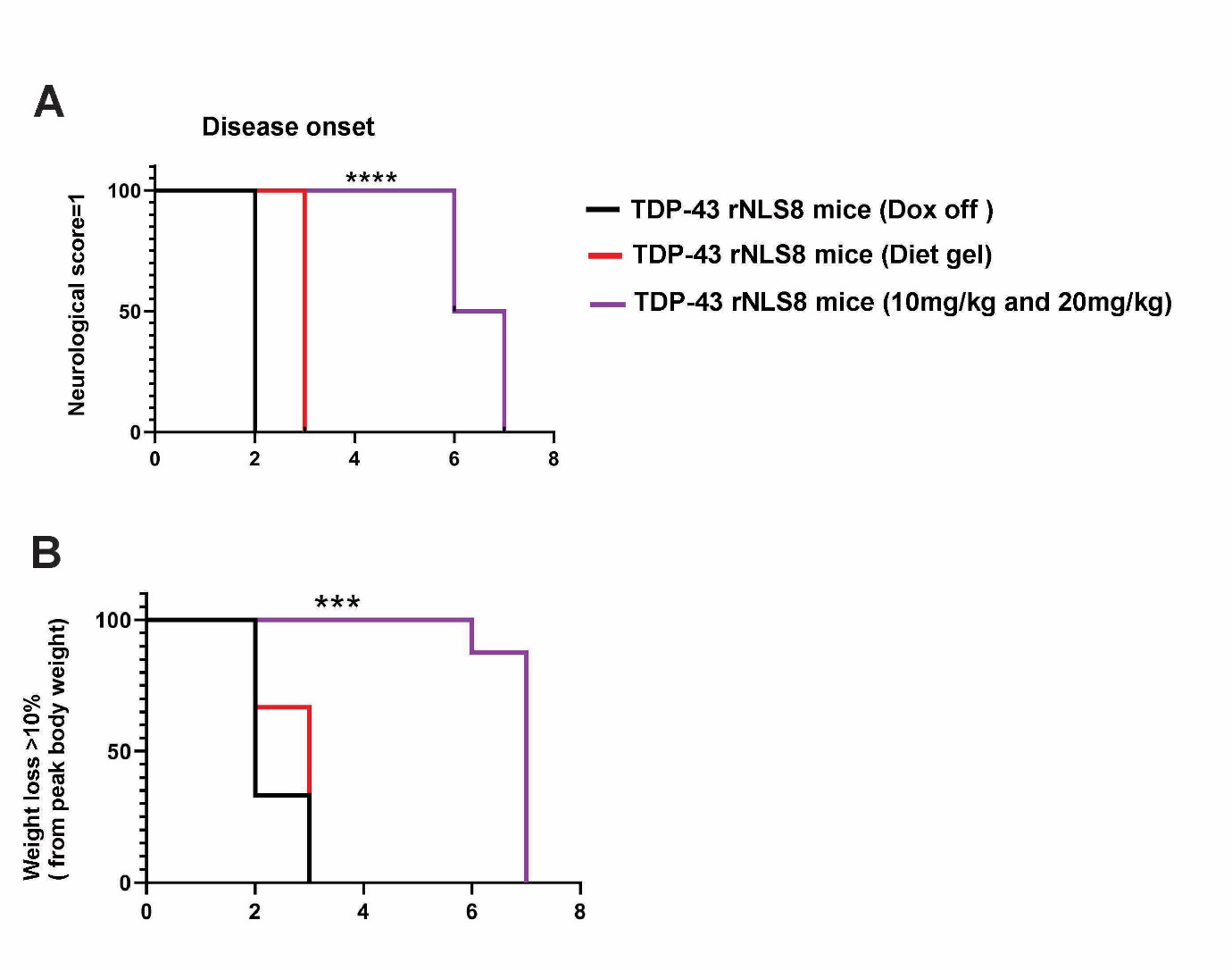
**

**(A)** Kaplan Meier curves representing age of disease onset in weeks, defined as an individual mouse achieving a neurological score =1 (NS=1). Log-rank (Mantel-Cox) test, *****p*<0.0001. Disease onset is delayed in mice supplemented with 10-20mg/kg Dox: mean = 7 weeks for combined 10mg/kg and 20mg/kg Dox group (n=8), compared to 2 weeks for Dox off (n=3), and 3 weeks for Diet gel groups (n=3). (**B)** Kaplan Meier curves representing age of disease onset in weeks, defined as an individual mouse achieving a weight loss of >10% from peak body weight. Log-rank (Mantel-Cox) test, ****p<*0.001. Disease onset is delayed in mice supplemented with 10-20mg/kg Dox: mean = 7 weeks for combined 10mg/kg and 20mg/kg Dox group (n=8), compared to 2 weeks for Dox off (n=3), and 3 weeks for Diet gel groups (n=3).

**Supplementary Figure 3**

**Combined analysis of disease progression of 10mg/kg and 20mg/kg Dox mice and controls (Dox off and Diet gel).**

**
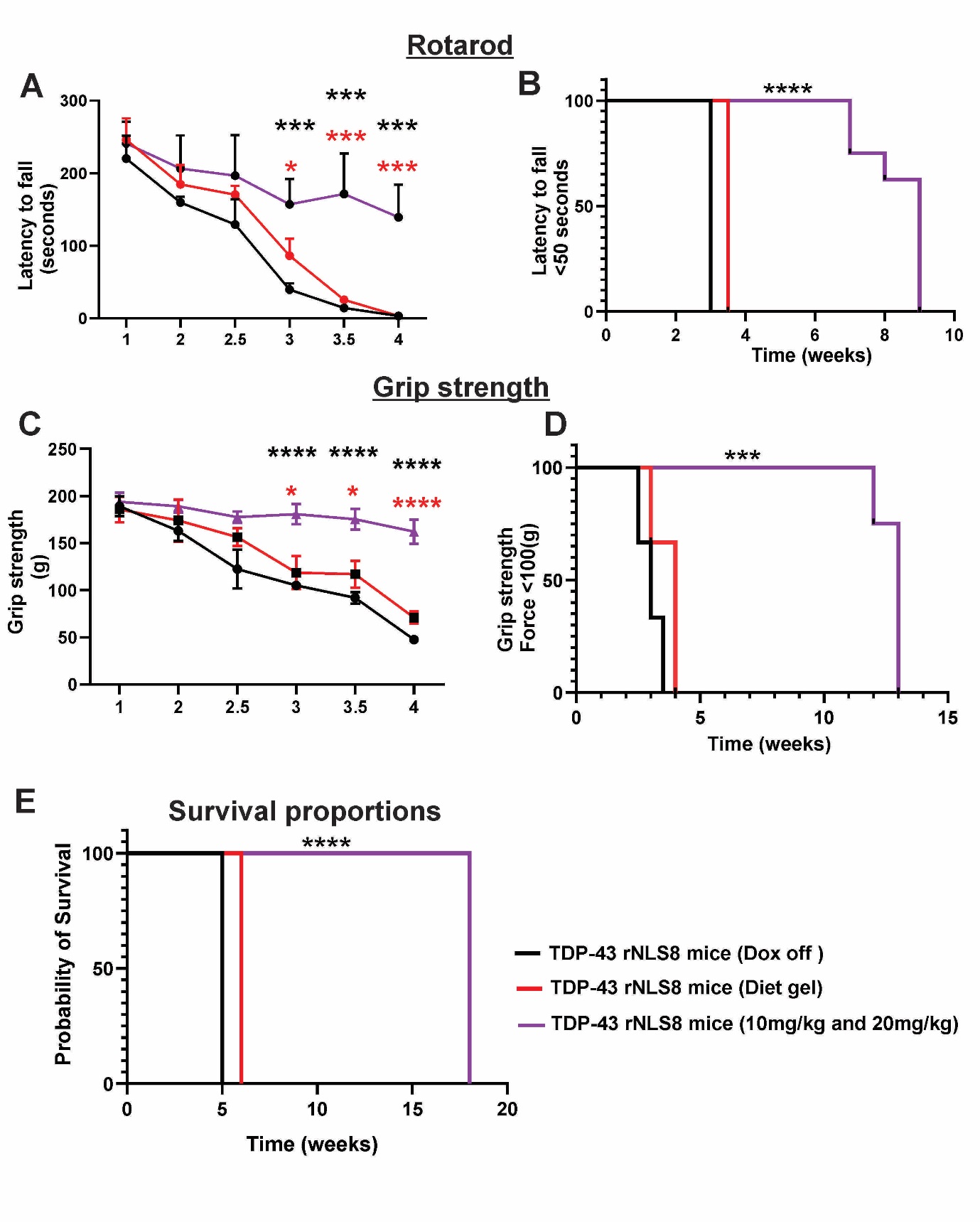
**

**(A)** Rotarod performance (latency to fall in seconds) is significantly improved at 3, 3.5 and 4 weeks in combined 10mg/kg and 20mg/kg Dox mice compared to Dox off and Diet gel animals, indicating improvement in motor function. Mixed effect analysis mean ± SD, two-way Anova, Tukey’s multiple test comparison, ****p*<0.001, **p*<0.05, n=3 (Dox off and Diet gel), n=8. (10mg/kg and 20mg/kg Dox). Black (*) and red (*) asterisks refer to comparisons to Dox off or Diet gel mice respectively. **(B)** Kaplan Meier curves representing Rotarod latency to fall, defined as an individual mouse latency to fall less than 50s. Log-rank (Mantel-Cox) test, *****p<0.0001*. With a median rotarod of 3 and 3.5 weeks for Dox off and Diet gel supplemented group (n=3) respectively, 9 weeks for 10mg/kg and 20mg/kg Dox plus Diet gel group (n=8). **(C)** Muscle function, indicated by grip strength, is significantly improved at 3, 3.5 and 4 weeks in combined 10mg/kg and 20mg/kg Dox mice compared to Dox off and Diet gel mice. Mixed effect analysis mean ± SD, two-way Anova, Tukey’s multiple test comparison, *****p<*0.0001, **p*<0.05, n=3 (Dox off and Diet gel group), n=8 (10mg/kg and 20mg/kg Dox group). Black (*) and red (*) asterisks refer to comparisons to Dox off or Diet gel mice respectively.  **(D)** Kaplan Meier curves representing grip strength, defined as an individual mouse achieving less than 100(g) force. Log-rank (Mantel-Cox) test, ****p<0.001*. With a median grip strength of 3 and 4 weeks for both dox off and Diet gel supplemented group (n=3), 13 weeks for 10mg/kg and 20mg/kg Dox plus Diet gel group(n=8)**. (E)** Kaplan Meier curves representing probability of survival, defined as an individual mouse achieving a neurological score =3 (NS=3). Log-rank (Mantel-Cox) test, *****p<*0.0001. Combined 10mg/kg and 20mg/kg Dox mice reach NS=3 at median = 18 weeks compared to 5 or 6 weeks for Dox off or Diet gel group respectively**.**
